# The Evolving Knowledge on T and NK Cells in Classic Hodgkin Lymphoma: Insights into Novel Subsets Populating the Immune Microenvironment

**DOI:** 10.3390/cancers12123757

**Published:** 2020-12-14

**Authors:** Isacco Ferrarini, Antonella Rigo, Carlo Visco, Mauro Krampera, Fabrizio Vinante

**Affiliations:** 1 Section of Hematology, Department of Medicine, University of Verona, 37134 Verona, Italy; antonella.rigo@univr.it (A.R.); carlo.visco@univr.it (C.V.); mauro.krampera@univr.it (M.K.); fabrizio.vinante@univr.it (F.V.); 2 Cancer Research and Cell Biology Laboratory, Department of Medicine, University of Verona, 37134 Verona, Italy

**Keywords:** classic Hodgkin lymphoma, microenvironment, T cells, NK cells, PD-1 blockade, immunotherapy

## Abstract

**Simple Summary:**

In classic Hodgkin lymphoma, T and NK cells constitute a significant fraction of the reactive microenvironment established by malignant Hodgkin Reed–Sternberg cells. Despite their abundance, T and NK cells remain largely ineffective because of two coordinated levels of immune evasion. The first is based on the acquisition of regulatory properties or exhausted phenotypes that cripple their antitumor activity. The second is represented by their peculiar spatial distribution, with the most immunosuppressive subpopulations lying in close proximity of neoplastic cells. Recent discoveries about the functional role and the spatial orientation of T and NK cells in classic Hodgkin lymphoma are the focus of this review.

**Abstract:**

Classic Hodgkin lymphoma (cHL) is a unique lymphoid neoplasm characterized by extensive immune infiltrates surrounding rare malignant Hodgkin Reed–Sternberg (HRS) cells. Different subsets of T and NK cells have long been recognized in the cHL microenvironment, yet their distinct contribution to disease pathogenesis has remained enigmatic. Very recently, novel platforms for high dimensional analysis of immune cells, such as single-cell RNA sequencing and mass cytometry, have revealed unanticipated insights into the composition of T- and NK-cell compartments in cHL. Advances in imaging techniques have better defined specific T-helper subpopulations physically interacting with neoplastic cells. In addition, the identification of novel cytotoxic subsets with an exhausted phenotype, typically enriched in cHL milieu, is shedding light on previously unrecognized immune evasion mechanisms. This review examines the immunological features and the functional properties of T and NK subsets recently identified in the cHL microenvironment, highlighting their pathological interplay with HRS cells. We also discuss how this knowledge can be exploited to predict response to immunotherapy and to design novel strategies to improve PD-1 blockade efficacy.

## 1. Introduction

**Figure 1:**
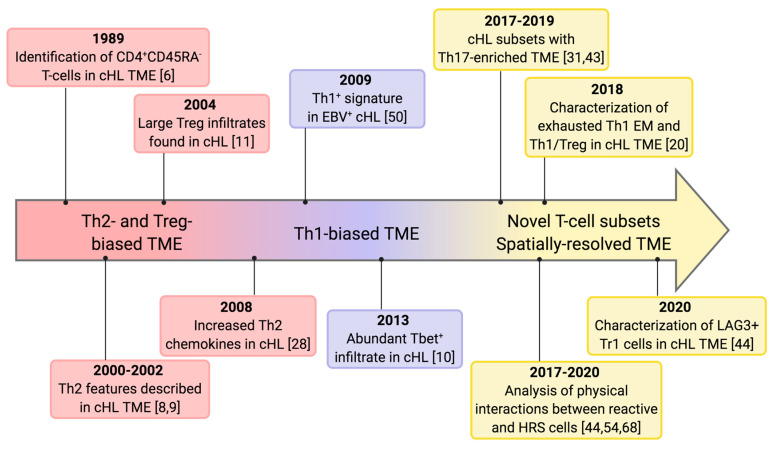
Timeline of T-cell infiltrates in cHL. Represented are the key milestones driving the understanding of T-cell infiltrates in cHL. The original view that the cHL tumor microenvironment (TME) is dominated by Th2 and Treg cells was challenged by evidences supporting the presence of numerous Th1 cells. More recently, the discovery of novel T-cell subsets and the analysis of their spatial relationships are adding further complexity to the cHL TME and may be useful to design novel microenvironment-based therapeutic strategies.

Classic Hodgkin lymphoma (cHL) accounts for 15% to 25% of all lymphomas, and is characterized by a bimodal distribution with an increased incidence among young adults and subjects of 55 years and older [1]. Traditional treatment approaches, combining chemotherapy and radiotherapy, have led to a high cure rate between 60% and 90%, mostly dependent on disease stage. Second line chemotherapy followed by autologous stem cell transplantation can rescue a proportion of relapsed/refractory (R/R) patients, but long-term toxicities still represent a major concern [1]. Since this disease was discovered [2], the unique features of its inflammatory microenvironment have intrigued generations of pathologists and clinicians, who devoted much energy to characterize the nature of immune cells and the reciprocal interactions between the neoplastic and reactive components. The malignant Hodgkin Reed–Sternberg (HRS) cells, which are distinguished into mononuclear (Hodgkin) and binucleated (Reed–Sternberg) forms [3,4] and constitute only a minor fraction (1–2%) of the overall tumor cellularity, are embedded within an extensive, yet functionally inefficient, immune infiltrate composed of lymphocytes, monocyte/macrophages, neutrophils, eosinophils, and NK cells [5]. T lymphocytes are the most abundant immune cells in the cHL milieu and encompass a variety of subpopulations with different localizations, multiple patterns of interaction with HRS cells and macrophages, and divergent functional properties [5]. Over the past thirty years, three distinct perspectives have alternatively dominated our understanding of cHL T-cell microenvironment (Figure 1). 

The initial evidence for the presence of a T helper (Th) 2-biased milieu in cHL was based on early immunohistochemistry data showing the majority of infiltrating T cells were CD45RO^+^CD45RA^-^CD45RB^low^ [6,7,8,9]. This pattern of expression was originally interpreted as a Th2-skewed phenotype because CD45 isoforms were thought to discriminate Th1/Th2 polarization, rather than T-cell differentiation status (naïve/antigen-exposed, see next section for more details) [6,7,10]. Moreover, the lack of high-throughput technologies limited the analysis to a few selected markers, potentially insufficient to capture the heterogeneity of the cHL microenvironment. Later, large infiltrates of regulatory T cells were identified in cHL and thought to be the major responsible for local immune evasion [11]. In 2013, flow cytometric analyses of single cell suspensions from cHL lymph nodes, together with tissue microarray immunohistochemistry, have challenged this view, showing the CD4^+^ infiltrate was mostly Th1 polarized and retained proliferative capacity and a cytokine-secretory phenotype as well [10]. More recently, mass cytometry [12], enabling the simultaneous detection of more than 40 markers in millions of individual cells, and single-cell RNA sequencing [13] have added tremendous insights into the cellular composition of cHL tissues, providing novel information about T and NK cells surrounding, and sometimes contacting, HRS cells. Two general paradigms are emerging. First, there is remarkable heterogeneity within reactive T-cell compartment of cHL nodes. Second, the spatial localization of T and NK cells, so far relatively neglected, may follow a histological hierarchy established by HRS cells, in which the most immunosuppressive subpopulations directly contact neoplastic cells and participate in the formation of the so-called immune-privileged niche. Such biological advances are being translated into the clinical ground, and are helping explain why cHL is one of the most sensitive to PD-1/PD-L1 axis inhibition among human cancers.

## 2. Overview of T-Cell Differentiation, Polarization and Activation Status

**Figure 2:**
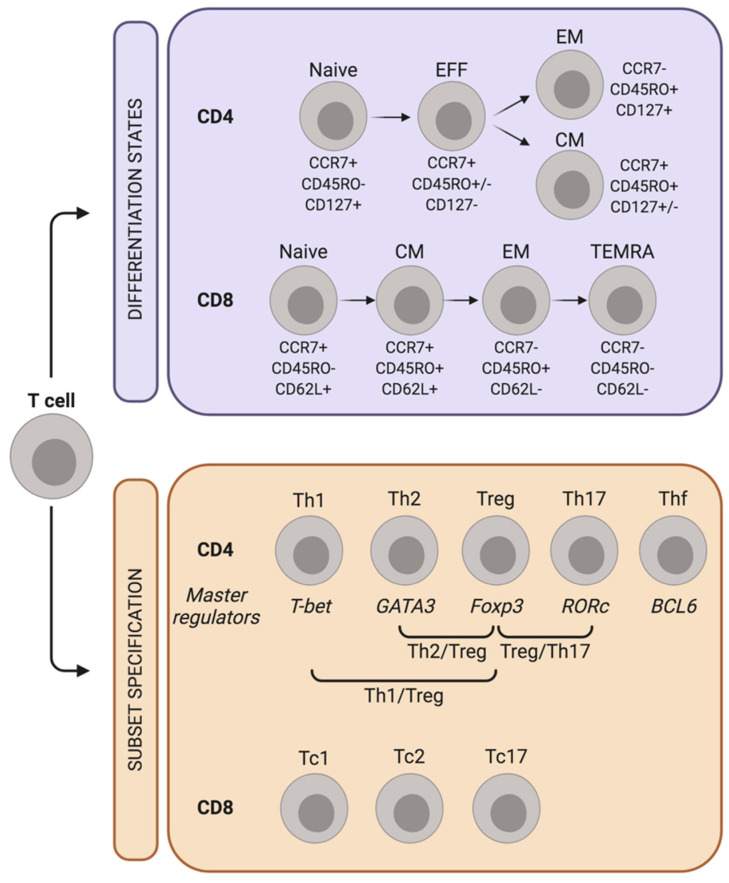
Differentiation and polarization of T cells. CD4^+^ T-cells differentiate from naïve to effectors (EFF), effector memory (EM) and central memory (CM) cells. CD8^+^ T-cells progress from naïve to CM, EM and terminal effector memory re-expressing CD45RA (TEMRA). CD4 and CD8 differentiation states can be distinguished by the expression of the indicated markers. In parallel, antigen-exposed CD4^+^ T-cells acquire one or more master regulators that specify the subset of functional polarization. Though less studied, a similar process seems to be operational also in CD8^+^ T-cells.

T cells are distinguished into two broad classes based on the expression of CD4 and CD8 co-receptors. CD4^+^ T-helper cells detect peptide antigens in the context of MHC class II (MHC-II) molecules and govern the adaptive arm of the immune response by producing cytokines and chemokines with either pro-inflammatory or regulatory roles. Instead, CD8^+^ cytotoxic T cells detect antigen presented by MHC class I (MHC-I) complex and elicit lytic responses that directly kill neoplastic or virus-infected cells [14]. Two parallel processes drive the peripheral fate of CD4^+^ and CD8^+^ T lymphocytes: cell differentiation and subset specification (Figure 2). 

T-cell differentiation states include naïve T cells, which have never encountered the antigen before, central memory (CM) T cells, effector memory (EM) T cells, and terminally differentiated T cells (TEMRA) [15]. Each of these clusters exhibits a distinct repertoire of surface receptors, and the analysis of CD45, CCR7, CD62L and CD95 is utilized for their proper identification [15]. The transition from naïve to terminally differentiated cells is characterized by a progressive change in the epigenetic and transcriptional profile, which parallels the acquisition of effector functions. Overall, an increased differentiation status positively correlates with the production of inflammatory and lytic molecules, but limits self-renewal capacity and longevity [16]. 

Subset specification allows T cells to specialize their response based on the cytokine environment they are exposed to. Most cytokines signal through the JAK/STAT pathway, with different members of the STAT family promoting functionally divergent T-cell subsets, and initiating the transcription of different T-cell master regulators [16]. STAT4 and STAT1, phosphorylated in response to IL12 or IFNγ, respectively, transactivate T-bet and switch cell polarization into a Th1 phenotype [17]. STAT5 and STAT6 define the Th2 subset by upregulating the transcription factor GATA3 [17]. Th17 polarization is induced by IL6 and IL23 via STAT3/RORc axis [18], whereas T-regulatory (Treg) differentiation is promoted by Foxp3 via STAT5 phosphorylation [19]. Although the lineage specification has been extensively studied in CD4^+^ T cells, cytotoxic T lymphocytes can be categorized similarly, and subsets producing type 1, type 2 cytokines, or even IL17 have been clearly identified and termed Tc1, Tc2 and Tc17, respectively [15]. 

As discussed below, the composition of T-cell compartment in cHL differs from that of reactive lymph nodes (RLNs), both in terms of differentiation state and subset specification [20]. Additionally, a more nuanced view of subset-defining transcription factors [16] should be considered while dissecting the cHL milieu, as populations co-expressing multiple transcriptional regulators have been increasingly described [20]. Because HRS cells release a variety of soluble factors involved in T-cell chemotaxis and polarization, they control almost every aspect of T cell biology in cHL tissue, spanning from recruitment to lineage polarization and functional exhaustion.

## 3. The Hodgkin Reed-Sternberg Secretome: Combinatorial Signals for T-Cell Recruitment

HRS cells release a vast array of cytokines and chemokines actively contributing to build up the unique cHL microenvironment [21]. 

CCL5 is a chemokine secreted by cHL cells upon CD40 stimulation, which is provided by CD4^+^CD40L^+^ T-lymphocytes rosetting HRS cells [22]. The CCL5 cognate receptor, CCR5, is usually expressed on circulating T cells, which migrate to neoplastic tissues following CCL5 gradients [23]. In addition, CCR5 is expressed on HRS cells and stimulates their proliferation upon interaction with CCL3, CCL4 and CCL5 [22]. Therefore, the CCL5/CCR5 axis provides autocrine and paracrine signals favoring tumor growth either through direct effects on cancer cells or enhancement of microenvironment formation. Indeed, CCR5 antagonism by maraviroc disrupts the interactions between tumor and reactive cells, impairing tumor xenograft growth without significant toxicity [24]. 

CCL11, also known as eotaxin, is secreted by cHL-associated fibroblasts following stimulation with HRS-derived TNFα [25]. Upon binding to CCR3, which is considered a Th2 marker [26], CCL11 promotes chemotactic responses of Th2 cells, suggesting that the stromal component of cHL lymph nodes can be educated by neoplastic cells to sustain the recruitment of immunosuppressive T-cell populations [25]. 

CCL17 (TARC) is another chemokine involved in Th2-cell recruitment. Van den Berg and colleagues showed the L428 cell line and several primary cHL tissues expressed CCL17, while anaplastic T-cell lymphomas and diffuse large B-cell lymphoma were mostly negative [27]. CCL17 binds to its receptor CCR4, usually detected on Th2 lymphocytes and responsible for their chemotactic migration [27]. CCL22 shares the same receptor as CCL17 and is also secreted by HRS cells. The concentration of both chemokines increases in sera from cHL patients with active disease and sharply decreases after treatment [28]. Mogamulizumab, an anti-CCR4 monoclonal antibody, exhibits clinical activity in CCR4^+^ systemic and cutaneous T-cell lymphomas, and may be of some benefit in contrasting the cHL microenvironment [29].

CCL20 (MIP3α) is a CC chemokine detected in a subset of cHL cell lines (L428, KM-H2, and L591) and primary tumors [30]. *CCL20* gene transcription is induced by the IL21/STAT3 axis, which is constitutively active in the cHL cell lines expressing both IL21 and IL21 receptor [30]. CCL20 recruits CCR6^+^ T lymphocytes, including Th17, Tregs, and a subset of Th1 [31]. CCR6^+^CD4^+^CD25^+^Foxp3^+^CD127^low^ regulatory cells are particularly enriched upon CCL20 stimulation and likely contribute to local immune evasion [30]. CCL20 expression in HRS cells can be also upregulated by the EBV-nuclear antigen 1 EBNA1 [32], indicating that EBV infection plays an active role in establishing the reactive microenvironment. In addition, EBV latent Membrane Protein 2A (LMP2A) induces *IL10* gene transcription by HRS cells through PI3K activation and STAT3 phosphorylation [33]. Because IL10 polarizes tumor-associated macrophages (TAMs) into the immunosuppressive, M2 phenotype [34], multiple EBV-related proteins end up blunting the anti-tumor potential of inflammatory cells. In accordance, elevated pre-treatment serum IL10 is associated with decreased progression-free survival (PFS) in cHL patients treated with either chemotherapy or radiation [35,36]. 

As reported by Machado and colleagues, several other T-cell homing molecules are detectable within the vasculature of cHL lesions. CCL21, CXCL10 and CXCL12 are expressed by HRS cells and/or vascular endothelium, while the cognate receptors CCR7, CXCR3 and CXCR4 are detected on a large proportion of T cells infiltrating the neoplastic tissue [37]. Moreover, HRS cell-derived lymphotoxin-α induces upregulation of adhesion molecules, such as ICAM-1, VCAM-1, and E-selectin, on vascular endothelium, further promoting T-cell recruitment into cHL lymph nodes [38]. Additional soluble factors attracting other components of the cHL microenvironment have been recently reviewed by Aldinucci et al [39]. 

## 4. The Spectrum of T-Helper Subsets in the cHL Microenvironment

T helper lymphocytes are the predominant T-cell population in cHL lymph nodes. A mass-cytometry-based, comparative analysis between RLNs and cHL cases revealed that Th1 effector memory (Th1 EM) and Th1 Treg were the main CD4^+^ subsets expanded in cHL tissues [20]. Th1 EM cells were defined as CCR7^-^CD45RO^+^EOMES^low^ effector lymphocytes co-expressing the Th1 transcription factor T-bet and the inhibitory co-receptor programmed death-1 (PD-1). As such, they likely represented an exhausted phenotype with low anti-tumor abilities, but potentially amenable to PD-1 blockade [20]. Th1 Tregs were CD25^+^Foxp3^+^ cells expressing T-bet but mostly PD-1-negative. This population had features associated with a memory phenotype (CCR7^low^Ki67^low^) and probably acts as a functionally active, immunosuppressive subset contributing to local immune evasion [20,40]. Therefore, a large proportion of the T-bet^+^ infiltrate turns out ineffective in cHL, owing to the peculiar coexistence of subpopulations functionally restrained by either PD-1 expression or regulatory cellular programs. 

From a genomic standpoint, the Treg infiltrates of cHL and reactive tonsils show remarkable differences [41]. cHL Tregs, defined by Wein et al. as CD4^+^CD25^high^CD127^−^, had higher levels of CD38 and autotaxin transcripts, involved in the modulation of purinergic signaling [41]. Moreover, the transcription of *BTLA* and *CD200R*, two genes encoding surface receptors with immunosuppressive functions, was upregulated by cHL Tregs. This may represent an additional immune-evasion mechanism as a subset of cHL cell lines expresses CD200 [41]. In vitro experiments also showed that Treg polarization was acquired by naïve CD4^+^ T-cells when co-cultured with cHL cell lines [41]. Unlike Th1 and Treg cells, the Th2 subsets are usually less represented in cHL tissues compared to RLNs. Th2 central memory (Th2 CM) and Th2 Treg cells are less abundant in cHL and their contribution in suppressing anti-lymphoma immunity is probably weaker than thought in the past [20,42]. In addition, they usually stain negative for PD-1, thus playing a minor role in the immune-checkpoint priming typically carried out by the PD-1^+^ Th1 subsets [20]. 

The extent of Th17 infiltrate in cHL is somehow more controversial, due to the different technical approaches across the studies (genomics, immunohistochemistry, mass cytometry), the different markers utilized for its identification, and the heterogeneity of cHL biology [20,31,43,44]. Overall, CD161^+^CCR4^+^ Th17 cells are less abundant in cHL than RLN [20]. However, about 40% of cHL cases display an IL17-enriched microenvironment, with histological evidence of IL17^+^ and even double positive Foxp3^+^IL17^+^ T cells, mostly located outside the close proximity of HRS cells [31]. Whether these subsets are counteracting the exhausted immune populations or rather represent another facet of cHL immune privilege is matter of ongoing investigations. Recently, a positive correlation between Treg/Th17 ratio and survival rate was shown in cHL patients [45], suggesting that large Th17 infiltrates may increase disease aggressiveness. Factors released by HRS, such as IL6, IL21, IL23, and soluble CD30 (sCD30) [46] can favor Th17 polarization [31,43,47,48], which, in turn, promotes the recruitment of myeloid cells and amplifies the inflammatory infiltrate [49]. Two independent, genomic-based analyses of the cHL microenvironment demonstrated the Th17-skewed immune profile was more common among EBV^−^ cases as compared to the positive ones [43,44]. Accordingly, the transcriptomic signature of EBV^−^ cHL showed the engagement of the IL23/IL17 axis, leading to STAT3 phosphorylation and nuclear translocation. By contrast, most EBV^+^ tumors were Th1-biased, with coordinate expression of T-bet, IFNγ and IDO genes [43,50]. In this context, the expansion of regulatory type 1 (Tr1) lymphocytes may be of substantial importance to allow HRS cells evading immune attack. Tr1 lymphocytes have been detected in both tumor tissue and peripheral blood of EBV^+^ cHL patients, and configure a regulatory subset lacking Foxp3, but usually expressing integrin subunit alpha 2 (ITGA2) and lymphocyte-activation gene 3 (LAG3) [51,52]. Tr1 cells produce high amount of the immunosuppressive cytokine IL10, which indeed is increased in the microenvironment as well as in serum samples of EBV^+^ cHL [52]. Therefore, Tr1 cells act as an additional source of IL10, which is also upmodulated by the latent EBV infection established in EBV^+^ HRS cells [33]. Very recently, Tr1 cells in the cHL milieu were further characterized and a distinct Foxp3^−^, Tr1-type T-cell subpopulation, staining positive for LAG3, CD25 and glucocorticoid-induced TNFR-related protein (GITR), has been identified irrespective of EBV positivity [44]. This LAG3^+^ cluster was significantly more evident in cHL cases compared with RLNs and its expansion was enhanced *in vitro* by culturing normal T cells with HRS cell supernatant. In particular, IL6 released by tumor cells played a major role in promoting LAG3^+^ cell polarization. Conversely, direct interaction with MHC-II, known to be a LAG3 ligand, led to LAG3 downmodulation, a mechanism accounting for the induction and persistence of LAG3^+^ cells in MHC-II-negative cHL cases [44]. Consistent with their immunosuppressive profile, LAG3^+^ cells expressed very high levels of IL10 and TGF-β, and impaired the proliferation of co-cultured CD4^+^ T-cells in vitro [44]. The LAG3^+^ subset in cHL also exhibited a peculiar repertoire of immunoreceptors, characterized by the expression of cytotoxic T-lymphocyte antigen 4 (CTLA4), which is considered a universal marker of regulatory T cells [53], and the lack of PD-1 [44]. As a consequence, LAG3^+^ cells represent an immunosuppressive population particularly enriched in MHC-II-negative cHL, and insensitive to antibodies targeting PD-1/PD-L1 axis. 

CTLA4 is expressed more broadly than LAG3 on T cells populating the cHL microenvironment [54]. Patel and colleagues have recently found that CTLA4^+^ T cells, usually staining negative for Foxp3, outnumbered PD-1^+^ and LAG3^+^ cells in cHL. Moreover, HRS cells and a fraction of TAMs were positive for the CTLA4 ligand CD86, whose engagement led to T-cell receptor (TCR) complex signaling inhibition [53]. Importantly, CTLA4^+^ T-cell infiltrate was further enriched in relapsed/refractory cHL patients, suggesting it might protect HRS cells from currently employed therapeutics, including anti-PD-1 antibodies [54]. 

## 5. Contribution and Localization of CD8^+^ T Cells in the cHL Microenvironment

Cytotoxic T cells are less abundant than Th cells in the cHL microenvironment, and their role in the pathogenesis of the disease has been recently downsized due to the low expression of β_2_-microglobulin and MHC-I on HRS cells in 79% of patients [55]. Because CD8^+^ T cells detect antigens in the context of MHC-I, HRS cells are invisible to the cytotoxic arm of T-cell immunity in a substantial proportion of cases. Nevertheless, in cHL cytotoxic T cells carry a peculiar differentiation profile, a biased polarization toward Tc1 phenotype, dysfunctional traits, and distinct intratumoral localization. Comparing to RLNs and tonsils, cHL tissues are enriched for EM and TEMRA CD8^+^ cells, whereas naïve and CM CD8^+^ cells are relatively fewer [20]. Together with a more differentiated profile, cHL cytotoxic T cells are also more Tc1-polarized and stain positive for granzyme B (GrB). By contrast, Tc2 cells are less frequent and overall less differentiated, with a prevalence of CM and a lack of EM Tc2 cells [20]. The increased terminal differentiation of cytotoxic T cells seems particularly evident in EBV^+^ cHL tumors, where MHC-I-dependent antigen presentation is more often intact [20,56]. EBV- and tumor-derived antigens persistently engage T-cell receptor and drive the development of dysfunctional states typically associated with a terminally-differentiated phenotype [57]. CD8^+^ dysfunction progresses along a gradient of cell states with different proliferation capacity and functional properties. While pre-dysfunctional cells are highly proliferating and express checkpoint inhibitors at intermediate levels, overtly dysfunctional CD8^+^ cells lose their proliferative thrust and increase the expression of checkpoints molecules such as CTLA4, LAG3 and PD-1 [58]. Traits of CD8 dysfunction are frequently encountered in cHL tissues. Multiplexed immunofluorescence imaging data have shown the density of CD8^+^CTLA4^+^ cells is higher in cHL cases compared with RLNs [54]. Moreover, most EM Tc1 cells express PD-1 [20], further highlighting the right-shift along the CD8^+^-cell dysfunctional axis [58]. An additional cytotoxic subset coexpressing CXCR5 and inducible T-cell costimulator (ICOS) has been recently identified in 7% of cHLs and still recapitulates the hallmarks of dysfunctional cells [59]. CD8^CXCR5+ICOS+^ cells have deficient cytotoxicity, secrete low levels of IFNγ, and support B-cell proliferation as well as antibody production in vitro [59]. These cells are frequently located in the close proximity of reactive B lymphocytes and produce high amounts of CXCL13, a B-cell chemoattractant responsible for the formation of tertiary lymphoid structures in several human cancers [60]. Indeed, the presence of a CD8^CXCR5+ICOS+^ population defines a cHL subset characterized by nodular architecture with hyperplastic germinal centers, only partly disrupted by the surrounding neoplastic infiltrate [59]. In addition to persistent antigenic stimulation, other soluble and membrane-bound molecules contribute to disempower CD8^+^ immunity in cHL. Galectin-1 is a member of a conserved family of carbohydrate-binding proteins frequently overexpressed by HRS cells. It induces apoptosis selectively in Th1 and cytotoxic T cells, thus favoring the secretion of Th2 cytokines, such as IL4, IL10 and IL13, and the expansion of regulatory T-cell subsets [61]. The abundance of galectin-1 together with the low MHC-I expression on HRS cells probably restrain CD8^+^ T lymphocytes to keep direct contact with neoplastic cells.

## 6. Natural Killer Cells in cHL: Keeping Cytotoxicity in Check 

NK cells are innate lymphoid cells exerting cytotoxic functions against malignant or virally infected cells. Their activity is regulated by the integration of signals derived from activatory and inhibitory receptors. Several killer-cell immunoglobulin-like receptors (KIRs), such as KIR2DL1, KIR2DL2/3, and KIR3DL1 transmit negative signals upon binding to MHC-I molecules, whereas neural cytotoxicity receptors (NCRs) and NKG2D respond to stress-induced stimuli and trigger NK cell activation [62]. Because the majority of cHL cases harbor inactivating mutations of β-2-microglobulin that lead to the loss of MHC-I expression [63], the functional profile of NK cells residing tumor microenvironment should be hypothetically skewed toward a persistent activation. However, independent studies have shown this is not the case. Soluble factors released by either HRS cells or infiltrating immune subpopulations can blunt NK cell recruitment and activation, leading to quantitative and qualitative NK deficiency [64]. The immunosuppressive cytokines IL10 and TGF-β, frequently abundant in the cHL milieu [11], reduce lymphocyte production of IFNγ, which would favor NK cell attraction [65]. In addition, HRS cells secrete soluble CD25 that binds IL2 and prevents its interaction with IL2R-expressing NK cells, limiting the availability of this pro-survival cytokine [66]. NK cell activation is further hindered by HRS overexpression of Fas ligand (FasL), which exerts a pro-apoptotic effect on Fas-expressing NK cells [67]. Even when NK populations resist the unfavorable cHL microenvironment, they are often excluded from the vicinity of HRS cells. A physical barrier consisting of immunosuppressive T and myeloid populations shields neoplastic cells from NK attack, a complementary and highly effective immune evasion strategy [68]. 

Beside these general, HRS-dependent, mechanisms of NK cell exclusion, recent studies have highlighted subtler differences in circulating NK subpopulations between cHL and healthy donors. Overall, NK cells can be distinguished into two main subsets identified as CD56^bright^CD16^−^ and CD56^dim^CD16^+^. While the former represents an immature NK phenotype and accounts for less than 10% of all NK cells in the circulation of healthy subjects, the latter is characterized by highly cytotoxic activity through lytic granule release and antibody-dependent cell cytotoxicity [69]. In cHL patients, the proportion of circulating CD56^bright^CD16^−^ NK subpopulation is significantly expanded as compared to healthy donors, and partially loses the expression of the chemokine receptor CCR7 [70]. This makes NK cells less prone to egress from the blood to the secondary lymphoid organs, other than less efficient in antibody-triggered cytotoxicity due to the lack of CD16 (FcγRIII). Monocyte-macrophages play a fundamental role in restraining NK cell activation, and monocyte depletion enhances the expression of the activation marker CD137 in cHL NK cells [70]. More conflicting results concern the expression of PD-1 in circulating NK cells. While Vari et al reported substantial PD-1 expression in CD56^bright^CD16^−^ NK cells [70], Cader and colleagues have demonstrated that PD-1 is largely absent in circulating NK cells [20], casting doubts about the role of PD-1/PD-L1 axis in blocking NK activity in cHL. 

Within the CD56^dim^ subpopulation, a novel subset staining negative for DNAX accessory molecule-1 (DNAM-1) has been recently identified [71]. CD56^dim^DNAM-1^−^ NK cells were enriched in the blood of cHL patients and displayed features of terminally differentiated NK cells such as propensity to undergo apoptosis in inflammatory microenvironments, resistance to cytokine withdrawal, and short telomeres. Compared with CD56^dim^DNAM-1^+^ NK cells, the CD56^dim^DNAM-1^−^ subset had lower proliferative potential, decreased ability to produce IFNγ in response to cytokine stimulation, and limited killing capacities. Additionally, CD56^dim^DNAM-1^−^ cells impaired the cytolytic activity of the CD56^dim^DNAM-1^+^ counterpart through undefined soluble factors, acting like an immunomodulatory subpopulation that further increases systemic immune evasion [71]. 

## 7. Immunosuppressive Interactions Taking Place in the Neoplastic Niche

**Figure 3:**
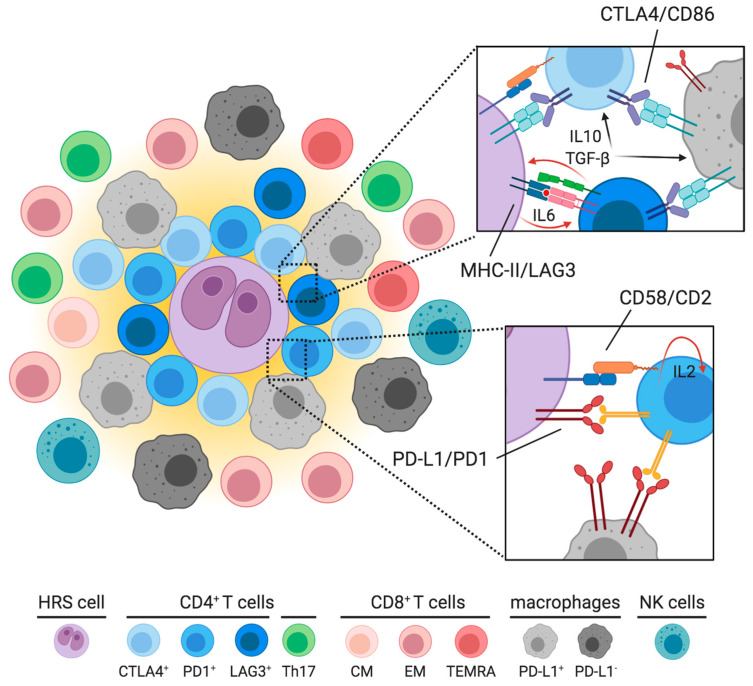
Preferential localizations of T-cell subpopulations in cHL. CD4^+^ T-cells localize in close proximity of HRS cells and form the typical rosettes. In particular, PD-1^+^ T-cells and CTLA4^+^ T-cells have been identified in contact with HRS cells and promote local immune evasion; LAG3^+^ T-cells have been described in the vicinity of HRS cells by some, but not all, authors. They are supported by HRS-derived IL6, and secrete IL10 and TGF-β that promote HRS survival and further suppress local immunity. CD58/CD2 interaction is critical for the establishment of the immunological synapse between HRS and CD4^+^ T-cells, and sustains the IL2-mediated autocrine signaling that leads to reactive T-cell expansion. Functional polarization of these checkpoint-defined T-cell subpopulations has yet to be determined, albeit Th1 subset should be the most abundant. Most of rosetting CD4^+^ T-cells participate in inhibitory interactions with HRS cells and PD-L1^+^ macrophages, which are also enriched nearby HRS cells. Outside the neoplastic niche, here represented by the yellow area surrounding the HRS cell, there are Th17 cells, PD-L1^-^ macrophages, CD8^+^ lymphocytes (mostly EM Tc1, with some variations depending on EBV status, see text) and a few NK cells. These potentially hostile populations are usually kept far away from neoplastic cells.

Despite the highly polymorphic and dynamic nature of cHL reactive microenvironment, the spatial localization of distinct immune subsets is all but random. Multiplexed immunofluorescence and imaging mass cytometry, enabling the simultaneous detection of multiple epitopes within a tissue section [72,73], have been exploited to unravel the cell-interaction network underlying cHL architecture. The emerging framework is that different reactive subpopulations occupy specific micro-areas and preferentially contact a few selected cell types (Figure 3). The HRS proximal region, also termed neoplastic niche and defined in two consecutive studies as the area within 75 μm of a given HRS cell [54,68], is usually enriched in PD-1^+^CD4^+^ T cells, which physically interact with both PD-L1^+^ cancer cells and PD-L1^+^ TAMs [68]. Additionally, the CD4^+^ population as a whole is more likely to be in close contact with HRS cells compared to the CD8^+^ T-cells, which show no enrichment around HRS cells [68]. The CTLA4^+^ T-regulatory subset is more represented in the HRS proximal region as well, and interacts with PD-L1^+^CD86^+^ TAMs [54]. Therefore, the immunosuppressive TAMs expressing PD-L1 and possibly CD86 are more frequently encountered in the close vicinity of HRS cells. This unique topology increases the local source of PD-L1 and likely augments the extent of PD-1 signaling. The localization of the CD4^+^Foxp3^+^ and CD4^+^LAG3^+^ T-cells remains instead more controversial [44,54], but it may be dictated by the expression of MHC-II on tumor cells. MHC-II^−^ cHL cases have a high density of LAG3^+^ T-cells in close proximity of HRS cells [44]. In this context, LAG3^+^ cells can also interact with other T-cell subsets and inhibit their proliferation and killing abilities. Conversely, MHC-II^+^ cases have a few LAG3^+^ T-cells, but display a greater number of Foxp3^+^ T-lymphocytes lying in the neoplastic niche close to HRS cells [44]. CD8^+^ T-cells, Th17 subsets, NK cells and PD-L1^−^CD86^low^ TAMs, potentially endowed with residual anticancer cytolytic activity, populate spatial domains that are further away from tumor cells [31,44,68]. Studies pointing out this unique cHL topology took into account primarily diagnostic samples, with scanty information about the spatial arrangement of relapsed tumors. Recently, Reinke S and colleagues have shown that cHL cases progressing after PD-1 blockade have a lower number of PD-L1^+^ macrophages with a slight increase of PD-1^+^ T-cells [74]. A previous study comparing diagnostic versus relapsed cHL demonstrated that also CTLA4^+^ T-cells are increased in relapsed cases [54]. This suggests that HRS cells surviving anti-PD1 treatment may be less dependent on the original microenvironment, or acquire different abilities to modulate such microenvironment. 

Overall, the variety of regulatory/exhausted T-cell populations and the highly organized topology of cHL tissues concur to establish a neoplastic niche with robust mechanisms of immune evasion. The interactions at the interface between HRS and rosetting CD4^+^ T-cells can be functionally categorized into two types. The first one is critical to stabilize CD4^+^ T-lymphocytes in the close proximity of HRS cells. It is represented by CD58/CD2 and, to a lesser extent, MHC-II/TCR interactions, which form an immunological synapse between neoplastic and T cells, and lead to the local accumulation of rosetting T-cells [75]. The second type of interactions serves to dampen T-cell antitumor activity and includes PD1/PD-L1, CTLA4/CD86 and LAG3/MHC-II interactions [76,77,78]. PD-1 engagement leads to phosphorylation of its immunoreceptor tyrosine-based switch motif and recruitment of SHP2, a phosphatase that inhibits ZAP70 and the co-stimulatory molecule CD28 in T cells [76]. The outcome is the attenuation of signaling branches derived from TCR and CD28 stimulation, including PI3K/AKT and RAS-MEK-ERK pathways [76]. Importantly, reverse signaling via PD-L1 can prevent apoptosis, stimulate mitochondrial oxidative phosphorylation, and favor proliferation of HRS cells in vitro [79], indicating that PD-1/PD-L1 axis can have direct pro-tumoral effects in addition to suppressing anti-tumor immunity. CTLA4 inhibits TCR via competition with CD28 for the ligands CD80 and CD86, for which CTLA4 has higher affinity and avidity. CTLA4 mitigates, therefore, the positive signals specifically originating from the co-stimulatory molecule CD28 [77]. Lastly, LAG3 binds MHC-II on HRS and antigen-presenting cells, with stronger affinity than CD4. As a consequence, LAG3 prevents the TCR complex/MHC-II interactions, directly dampening TCR signaling and impairing T-cell proliferation and cytokine production [78]. 

## 8. How HRS, T and NK Cells Predict Response to PD-1 Blockade

Until 2015, therapy of cHL was mostly directed to the neoplastic component. Although combination chemotherapy, radiation, and anti-CD30 immunoconjugates yielded successful results, a small but significant fraction of cHL patients still died of their disease [1]. In 2015–2016, the results of two phase 1 trials testing the anti-PD-1 antibodies nivolumab and pembrolizumab showed highly encouraging response rates with acceptable safety profile in heavily pre-treated populations [80,81], opening the way to novel, microenvironment-directed strategies in the management of this disease. The results of the phase 2 trials Checkmate 205 (nivolumab) and KEYNOTE-087 (pembrolizumab) involving R/R cHL patients showed an overall response rate (ORR) of 71% and 72%, with complete remission (CR) achieved in 21% and 28% of patients, respectively. The median duration of response was 18 months in the Checkmate 205 trial and 16.5 months in the KEYNOTE-087 [82,83]. 

With the aim of identifying biomarkers of response to PD-1 blockade, fluoresce in situ hybridization (FISH) assay was performed to characterize copy number alterations of PD-L1 in biopsy specimens from cHL patients. Ninety-seven per cent of cHL cases had abnormalities in the PD-L1 and PD-L2 loci, including polysomy (5% of cases), copy gain (56%), and amplification (36%) (55,84). The same amplicon at 9p24.1 almost always includes the *JAK2* gene, leading to increased JAK/STAT signaling in HRS cells and further induction of PD-L1 expression [84]. Constitutively active JAK/STAT pathway also promotes IL6, IL13 and CCL17 synthesis, thus contributing to recruit and polarize reactive cells [85]. Patients with higher-level 9p24.1 copy gain and increased expression of PD-L1 specifically on HRS cells had superior PFS after nivolumab treatment. Moreover, while MHC-I expression did not predict clinical outcome, MHC-II positivity on HRS cells was associated with better chances to achieve a CR [84]. This suggests that the CD4^+^ infiltrate rather than the CD8^+^ one might be the major determinant of the therapeutic activity of PD-1 inhibitors. Indeed, studies on murine solid tumor models revealed that PD-1 blockade exerted anticancer effects against MHC-I^−^MHC-II^+^ but not MHC-I^−^MHC-II^−^ tumors. In addition, the anticancer activity of anti-PD-1 antibodies was abolished in mice depleted of CD4^+^ T-cells, but not CD8^+^ T-cells [86]. Independent studies have also shown that Th lymphocytes may mount a cytolytic response in appropriate contexts [86,87]. Mice bearing MHC-II^+^ tumors had larger amounts of intratumoral CD4^+^ T-cells that stained positive for GrB, and this subset further increased following PD-1 blockade [86]. Similarly, a candidate circulating CD4^+^ “cytotoxic” T-cell subset (GrB^+^PD-1^+^ with EM phenotype) has been identified in cHL patients, particularly in the R/R setting. A CD4^+^ GrB^+^, but CD3^−^, innate subpopulation was also detected both in the circulation and in the tumor microenvironment of relapsed cHL cases, and was associated with a favorable response to nivolumab [87]. 

Additional factors potentially predicting good outcome following checkpoint inhibitors are T-cell diversity and NK cell abundance. In comparison with healthy subjects, cHL patients, especially those with R/R disease, have a significantly reduced TCR repertoire. In the Checkmate 205 trial, treatment with nivolumab was more effective in patients having a diverse peripheral TCR repertoire and an expansion of singleton T-cell clones during treatment. Patients with a highly significant increase in CD4^+^, but not CD8^+^, TCR diversity after 6 weeks of PD-1 inhibition were more likely to achieve a CR. Likewise, patients with more abundant circulating mature NK cells (CD56^dim^CD16^+^) had better clinical outcome after nivolumab [87].

## 9. Translational Insights to Improve Immunotherapy Results

**Figure 4:**
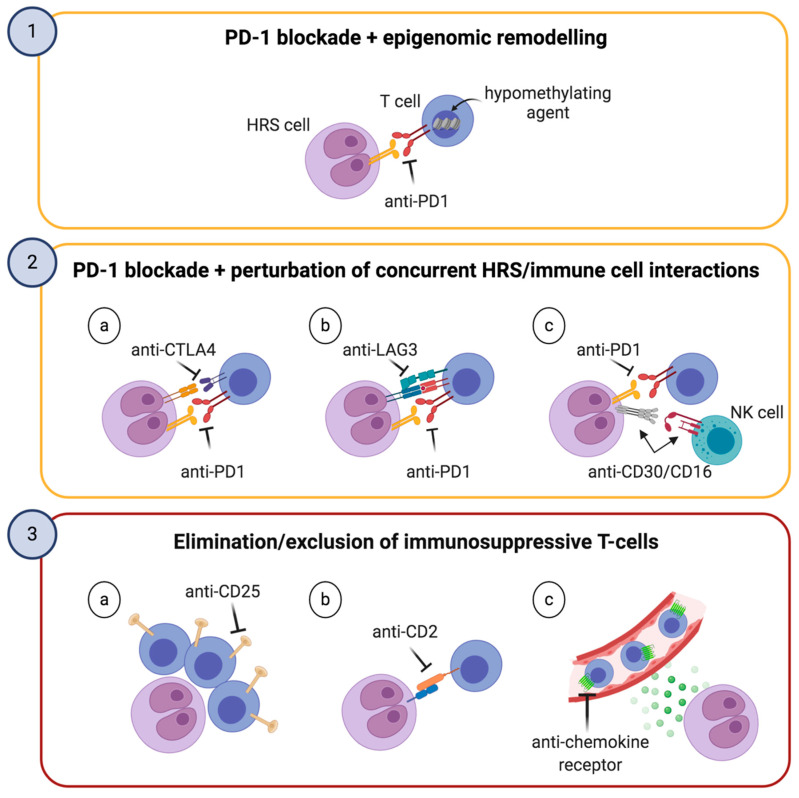
Three microenvironment-based strategies to improve PD-1 blockade or as alternatives thereof. The first and second boxes, outlined in yellow, illustrate two different strategies to improve the efficacy of PD-1 blockade. These include the combination with hypomethylating agents, which can reinvigorate exhausted T-cells, and the simultaneous perturbation of concurrent HRS/immune cell interactions. The third box, outlined in red, depicts alternatives to PD-1 blockade potentially useful for patients without biological predictors of response to checkpoint inhibitors or after their failure.

In general, cHL holds the highest reported responses to PD-1 inhibition in any tumor type [82,83], likely because of its genetically driven PD-L1 upregulation, the high mutational burden, the high frequency of MHC-II expression on HRS cells, the CD4^+^-enriched microenvironment and the abundance of PD-L1^+^ TAMs. The combination of these features renders cHL a lymphoid neoplasm uniquely primed for PD-1 blockade [88]. However, the robustness of complementary immune-evasion mechanisms typical of cHL (Figure 3) may be responsible for the primary and acquired resistance to anti-PD-1 treatment, as well as for the low rate of CRs. Upregulation of alternative immune checkpoints has already been associated with adaptive resistance to anti-PD-1 agents in solid tumors [89]. Therefore, novel strategies aiming at potentiating the effect of PD-1 blockade or switching to different microenvironment-targeting agents are worth being tested in cHL (Figure 4). 

One of the most promising approaches consists in combining PD-1 blockade with low-dose hypomethylating agents (Figure 4, upper panel). Pre-clinical in vivo studies have reported that reinvigoration of exhausted CD8^+^ T-cells (T_EX_) is only temporary after PD-1/PD-L1 axis inhibition due to the limited epigenetic flexibility of T_EX_ [90,91]. Essentially, T_EX_ fail to remodel their epigenetic landscape upon treatment with anti-PD-1 antibodies, preventing the acquisition of a memory phenotype. Thus, the persistence of HRS-derived antigenic stimulation can cause T_EX_ to enter a “re-exhausted” phenotype, with low anti-tumor activity. Based on the hypothesis that epigenetic modifiers may improve the long-term durability of T-cell reinvigoration, a phase II trial testing the combination of decitabine (a hypomethylating drug currently approved for myelodysplastic syndrome and acute myeloid leukemia) and camrelizumab (a fully humanized anti-PD-1 antibody) has been conducted [92]. In R/R patients who were anti-PD-1 naïve, CR rate with the combination therapy doubled as compared to camrelizumab alone (71% vs. 32%), and the response duration rate at 6 months was 100% for the combination versus 76% for camrelizumab alone [92]. Though encouraging, these results need to be validated in larger clinical trials. Moreover, because the efficacy of PD-1 blockade in cHL mainly depends on CD4/MHC-II rather than CD8/MHC-I system [84], it seems reasonable that additional biological mechanisms may underlie the synergism between hypomethylating drugs and PD-1 blockade. 

A second way to enhance immunotherapy might be the simultaneous perturbation of multiple immune interactions (Figure 4, middle panel). As discussed earlier, CD4^+^ T-cells expressing the inhibitory co-receptor CTLA4 are particularly abundant in cHL, frequently contact HRS cells, and are further enriched after PD-1 blockade [54]. A phase 1b trial has been conducted to address if the combination of nivolumab plus ipilimumab, an anti-CTLA4 antibody, could be safe and possibly improve clinical responses compared to nivolumab alone. Disappointingly, the ORR and CR rate for the combination (74% and 23%, respectively) were similar to those previously reported for nivolumab alone. In addition, the toxicity of the combination was higher than expected from single-agent nivolumab [93]. Despite this, future clinical investigations may define patient subsets particularly benefiting from this combination. At least in solid tumors, anti-CTLA4 treatment requires intact MHC-I system to effectively exert antitumor activity [94]. Therefore, it is tempting to speculate that ipilimumab ± nivolumab might confer a clinical advantage in the minor fraction of cHL patients with low/absent MHC-II but conserved MHC-I expression. Beside PD-1 and CTLA4, LAG3 is an additional targetable checkpoint. Several anti-LAG3 antibodies have being developed and some of them are in early-phase clinical trials for R/R lymphomas (NCT03489369, NCT03005782, NCT02061761). A preclinical study by Burova and colleagues showed that REGN3767, a fully human antibody targeting LAG3, increased the efficacy of PD-1 blockade in a humanized mouse model and enhanced the production of pro-inflammatory cytokines by tumor-specific T lymphocytes [95]. Similarly, combining PD-1 blockade and the anti-LAG-3 antibody LBL-007 resulted in more effective control of tumor growth than the single agents alone [96]. Preliminary data from a phase 1/2 study in melanoma patients showed the combination of nivolumab with BMS-986016 (IgG4 antibody targeting LAG3) had similar safety profile to nivolumab monotherapy, with encouraging efficacy [97]. Modulating HRS-NK interactions to unleash NK cell cytolysis is being explored and combined with PD-1 blockade. AFM13 is a CD30/CD16A bispecific antibody acting as an innate immune engager. It creates a bridge between CD30, expressed on HRS cells, and the activating receptor CD16A, expressed on NK cells and to a lesser extent on macrophages and γ/δ T cells. A phase 1 dose-escalation study showed AFM13 monotherapy was well-tolerated and active in brentuximab vedotin-refractory patients. Additionally, AFM13 resulted in significant NK-cell activation and reduction of sCD30 in peripheral blood [98]. A subsequent phase 1b trial testing the combination of AFM13 with pembrolizumab demonstrated that the combination had similar safety profile compared to each agent alone, with a promising ORR of 83% [99]. Therefore, CD16A engagement might restore anticancer cytotoxic properties in functionally-deficient innate immune cells, possibly synergizing with PD-1 blockade. It remains undefined, however, the exact mechanism whereby distant cells such as the NK population can be properly re-oriented to the HRS neoplastic niche. 

A third strategy might consist in either eliminating immunosuppressive T-cell populations or preventing their recruitment to the cHL microenvironment (Figure 4, lower panel). This approach might represent an attractive alternative for patients lacking predictors of good response to checkpoint inhibition, or after the failure thereof. In this context, CD25 is a convenient target because of its broad expression on CD4^+^ Treg lymphocytes rosetting HRS cells [20]. ^90^Y-daclizumab is a radiolabeled anti-CD25 antibody directed towards Treg cells in the cHL milieu. Its anticancer activity is due to strong β emissions that kill both the tumor-supporting lymphocytes and, via crossfire effect, the HRS cells nearby. Forty-six patients with R/R cHL were administered with up to seven intravenous infusions of ^90^Y-daclizumab. Of those, 23 patients underwent clinical improvement, with 14 CR and 9 partial responses (PR) [100]. ADCT-301 (camidanlumab tesirine) is another CD25-targeting antibody, conjugated to a pyrrolobenzodiazepine dimer toxin. Interim data of a phase 1 clinical study involving heavily-pretreated cHL patients treated with ADCT-301 showed an ORR of 63%, with a CR rate of 27% [101]. However, final outcomes have not been released yet. Eliminating rosetting T-cells by targeting the HRS/T immunological synapse might be an additional manner to deprive the neoplastic niche of immunosuppressive populations. Because the CD2/CD58 interaction stabilizes the HRS/T synapses [75], monoclonal anti-CD2 antibodies, already in clinical trials for T-cell lymphomas and autoimmune conditions [102], may counter rosette formation and prove of some benefit in cHL. Lastly, recruitment of tumor-nurturing immune subsets can be blocked by acting on chemokine/chemokine receptor pairs. In this regard, the CCR5-antagonist maraviroc, already licensed by the US Food and Drug administration for HIV treatment, may be repurposed for cHL based on pre-clinical data showing inhibition of monocyte recruitment and shrinkage of tumor masses in vivo [24]. As previously mentioned, the anti-CCR4 antibody mogamulizumab, which is being tested in T-cell lymphomas, might similarly counteract microenvironment formation and prove of therapeutic relevance in selected cHL patients [29].

In addition to these microenvironment-based strategies, HRS-directed therapies may potentiate PD-1 blockade as well. In particular, regimens based on the combination of nivolumab with the anti-CD30 immunoconjugate brentuximab vedotin have recently been tested and may represent an effective chemo-free option for R/R patients or for those unsuitable for chemotherapy [103]. Interestingly, CD30 can be released by HRS cells either through the shedding of its ectodomain (sCD30) or loaded on extracellular vesicles (EV). sCD30 alters Th1/Th17 balance [31] while CD30-loaded EV can be absorbed on reactive immune cells that become potential targets of anti-CD30-based immunotherapy [104]. Therefore, brentuximab vedotin and anti-CD30 CAR-T cells [105] may have yet poorly defined immunomodulatory roles in addition to their direct effects against tumor cells.

## 10. Concluding Remarks

The data reviewed herein point to a meticulous arrangement of the environment surrounding HRS cells, potentially actionable with an increasing number of targeted agents. Spatially-resolved immunologic studies have enabled to identify niches of immunoprotection where HRS cells are sheltered by a double layer of reactive cells: *a)* the CD4^+^ infiltrate, composed of exhausted PD-1^+^ T cells, active Tregs, and additional CTLA4^+^ and LAG3^+^ subsets, whose abundance may depend on disease status (treatment-naïve vs. R/R) and MHC-II expression; *b)* the PD-L1^+^ macrophages, likely polarized by the cytokine milieu. Although peculiar genetic bases and a primed microenvironment put cHL at the top of the PD-1-blockade-sensitive human cancers, important questions remain to be addressed. Indeed, the effector mechanisms elicited by the CD4^+^ T cells following anti-PD-1 treatment, and whether HRS cells are sensitive to CD8^+^ T-cell reinvigoration, remain to be determined. Moreover, the immune environment of cHL may change in relation to disease status and prior therapies. Now that PD-1 blockade is increasingly used in clinical practice, it would be valuable to understand the impact of temporally-distinct microenvironments on checkpoint inhibitor activity. Future research will shed further light on the immune architecture around HRS cells, with the hope to maximize the results of immune-oncology manipulation in cHL. R/R cases, treatment-naïve patients with unfavorable prognostic factors, and elderly patients unsuitable for chemotherapy may particularly benefit from PD-1 blockade-based strategies in the near future. 

## References

[1] Connors J.M., Cozen W., Steidl C., Carbone A., Toppe R.T., Flechtner H.H., Bartlett N.L. (2020). Hodgkin lymphoma. Nat. Rev. Dis. Primers.

[2] Hodgkin T. (1832). On some morbid appearances of the absorbent glands and spleen. Med. Chir. Trans..

[3] Reed D. (1902). On the pathological changes in Hodgkin’s disease with special reference to its relation to tuberculosis. John Hopkins Hosp. Rep..

[4] Sternberg C. (1898). Über eine eigenartige unter dem Bilde der Pseudoleukämie verlaufende Tuberkolose des lymphatischen Apparates. Z. Heilkd..

[5] Calabretta E., d’Amore F., Carlo-Stella C. (2019). Immune and Inflammatory Cells of the Tumor Microenvironment Represent Novel Therapeutic Targets in Classical Hodgkin Lymphoma. Int. J. Mol. Sci..

[6] Poppema S. (1989). The Nature of the Lymphocytes Surrounding Reed-Sternberg Cells in Nodular Lymphocyte Predominance and in Other Types of Hodgkin’s Disease. Am. J. Pathol..

[7] Poppema S., Potters M., Visser L., van den Berg A.M. (1998). Immune escape mechanisms in Hodgkin’s disease. Ann. Oncol..

[8] Poppema S., van den Berg A.M. (2000). Interaction between host T cells and Reed–Sternberg cells in Hodgkin lymphomas. Semin. Cancer Biol..

[9] Skinnider B.F., Mak T.W. (2002). The role of cytokines in classical Hodgkin lymphoma. Blood.

[10] Greaves P., Clear A., Owen A., Iqbal S., Lee A., Matthews J., Wilson A., Calaminici M., Gribben J.B. (2013). Defining characteristics of classical Hodgkin lymphoma microenvironment T-helper cells. Blood.

[11] Marshall N.A., Christie L.E., Munro L.R., Culligan D.J., Johnston P.W., Barker R.N., Vickers M.A. (2004). Immunosuppressive regulatory T cells are abundant in the reactive lymphocytes of Hodgkin lymphoma. Blood.

[12] Gadalla R., Noamani B., MacLeod B.L., Dickson R.J., Guo M., Xu W., Lukhele S., Elsaesser H.J., Razak A.R.A., Hirano N. (2019). Validation of CyTOF Against Flow Cytometry for Immunological Studies and Monitoring of Human Cancer Clinical Trials. Front. Oncol..

[13] Yu X., Zhang L., Chaudhry A., Rapaport A.S., Ouyang W. (2020). Unravelling the heterogeneity and dynamic relationships of tumor-infiltrating T cells by single-cell RNA sequencing analysis. J. Leuk. Biol..

[14] Fang P., Li X., Dai J., Cole L., Camacho J.A., Zhang Y., Ji Y., Wang J., Yang X.F., Wang H. (2018). Immune cell subset differentiation and tissue inflammation. J. Hematol. Oncol..

[15] Kishton R.J., Sukumar M., Restifo N.P. (2017). Metabolic Regulation of T Cell Longevity and Function in Tumor Immunotherapy. Cell Metab..

[16] Vahedi G., Poholek A.C., Hand T.W., Laurence A., Kanno Y., O’Shea J.J., Hirahara K. (2013). Helper T-cell identity and evolution of differential transcriptomes and epigenomes. Immunol. Rev..

[17] Zhu J., Yamane H., Paul W.E. (2010). Differentiation of effector CD4 T cell populations. Annu. Rev. Immunol..

[18] Peters A., Lee Y., Kuchroo V.K. (2011). The many faces of Th17 cells. Curr. Opin. Immunol..

[19] Murawski M.R., Litherland S.A., Clare-Salzler M.J., Davoodi-Semiromi A. (2006). Upregulation of Foxp3 Expression in Mouse and Human Treg Is IL-2/STAT5 Dependent: Implications for the NOD STAT5B Mutation in Diabetes Pathogenesis. Ann. N. Y. Acad. Sci..

[20] Cader F.Z., Schackmann R.C.J., Hu X., Wienand K., Redd R., Chapuy B., Ouyang J., Paul N., Gjini E., Lipschitz M. (2018). Mass cytometry of Hodgkin lymphoma reveals a CD4+ regulatory T-cell-rich and exhausted T-effector microenvironment. Blood.

[21] Pizzolo G., Vinante F., Chilosi M., Romagnani S., Del Prete G. (1994). CD30 Antigen and Cellular Biology of Reed-Sternberg Cells. Blood.

[22] Aldinucci D., Lorenzon D., Cattaruzza L., Pinto A., Gloghini A., Carbone A., Colombatti A. (2008). Expression of CCR5 receptors on Reed–Sternberg cells and Hodgkin lymphoma cell lines: Involvement of CCL5/Rantes in tumor cell growth and microenvironmental interactions. Int. J. Cancer.

[23] Machicote A., Belén S., Baz P., Billordo L.A., Fainboim L. (2018). Human CD8+ HLA-DR+ Regulatory T Cells, Similarly to Classical CD4+FoxP3+ cells suppress Immune Responses via PD-1/PD-L1 Axis. Front. Oncol..

[24] Casagrande N., Borghese C., Visser L., Mongiat M., Colombatti A., Aldinucci D. (2019). CCR5 antagonism by maraviroc inhibits Hodgkin lymphoma microenvironment interactions and xenograft growth. Haematologica.

[25] Jundt F., Anagnostopoulos I., Bommert K., Emmerich F., Müller G., Foss H.D., Royer H.D., Stein H., Dörken B. (1999). Hodgkin/Reed-Sternberg Cells Induce Fibroblasts to Secrete Eotaxin, a Potent Chemoattractant for T Cells and Eosinophils. Blood.

[26] Kong S., Kim B.S., Uhm T.G., Lee W., Lee G.R., Park C.S., Lee C.H., Chung I.Y. (2013). Different GATA Factors Dictate CCR3 Transcription in Allergic Inflammatory Cells in a Cell Type-Specific Manner. J. Immunol..

[27] van den Berg A., Visser L., Poppema S. (1999). High Expression of the CC Chemokine TARC in Reed-Sternberg Cells. A possible explanation for the characteristic T-cell infiltratein Hodgkin’s lymphoma. Am. J. Pathol..

[28] Niens M., Visser L., Nolte I.M., van der Steege G., Diepstra A., Cordano P., Jarrett R.F., Te Meerman G.J., Poppema S., van den Berg A. (2008). Serum chemokine levels in Hodgkin lymphoma patients: Highly increased levels of CCL17 and CCL22. Br. J. Haematol..

[29] Ogura M., Ishida T., Hatake K., Taniwaki M., Ando K., Tobinai K., Fujimoto K., Yamamoto K., Miyamoto T., Uike N. (2014). Multicenter Phase II Study of Mogamulizumab (KW-0761), a Defucosylated Anti-CC Chemokine Receptor 4 Antibody, in Patients with Relapsed Peripheral T-Cell Lymphoma and Cutaneous T-Cell Lymphoma. J. Clin. Oncol..

[30] Lamprecht B., Kreher S., Anagnostopoulos I., Jöhrens K., Monteleone G., Jundt F., Stein H., Janz M., Dörken B., Mathas S. (2008). Aberrant expression of the Th2 cytokine IL-21 in Hodgkin lymphoma cells regulates STAT3 signaling and attracts T reg cells via regulation of MIP-3alpha. Blood.

[31] Ferrarini I., Rigo A., Zamò A., Vinante F. (2019). Classical Hodgkin lymphoma cells may promote an IL-17-enriched microenvironment. Leuk. Lymphoma.

[32] Baumforth K.R.N., Birgersdotter A., Reynolds G.M., Wei W., Kapatai G., Flavell J.R., Kalk E., Piper K., Lee S., Machado L. (2008). Expression of the Epstein-Barr Virus-Encoded Epstein-Barr Virus Nuclear Antigen 1 in Hodgkin’s Lymphoma Cells Mediates Up-Regulation of CCL20 and the Migration of Regulatory T Cells. Am. J. Pathol..

[33] Incrocci R., Barse L., Stone A., Vagvala S., Montesano M., Subramaniam V., Swanson-Mungerson M. (2017). Epstein-Barr Virus Latent Membrane Protein 2A (LMP2A) enhances IL-10 production through the activation of Bruton’s tyrosine kinase and STAT3. Virology.

[34] Murray P.J., Allen J.E., Biswas S.K., Fisher E.A., Gilroy D.W., Goerdt S., Gordon S., Hamilton J.A., Ivashkiv L.B., Lawrence T. (2014). Macrophage Activation and Polarization: Nomenclature and Experimental Guidelines. Immunity.

[35] Sarris A.H., Kliche K.O., Pethambaram P., Preti A., Tucker S., Jackow C., Messina O., Pugh W., Hagemeister F.B., McLaughlin P. (1999). Interleukin-10 levels are often elevated in serum of adults with Hodgkin’s disease and are associated with inferior failure-free survival. Ann. Oncol..

[36] Visco C., Vassilakopoulos T.P., Kliche K.O., Nadali G., Viviani S., Bonfante V., Medeiros L.J., Notti P., Rassidakis G.Z., Peethambaram P. (2004). Elevated Serum Levels of IL-10 are Associated with Inferior Progression-Free Survival in Patients with Hodgkin’s Disease Treated with Radiotherapy. Leuk. Lymphoma.

[37] Machado L., Jarrett R., Morgan S., Murray P., Hunter B., Hamilton E., Crocker J., Thomas W., Steven N., Ismail T. (2009). Expression and function of T cell homing molecules in Hodgkin’s lymphoma. Cancer Immunol. Immunother..

[38] Fhu C.W., Graham A.M., Yap C.T., Al-Salam S., Castella A., Chong S.M., Lim Y.C. (2014). Reed-Sternberg cell-derived lymphotoxin-a activates endothelial cells to enhance T-cell recruitment in classical Hodgkin lymphoma. Blood.

[39] Aldinucci D., Borghese C., Casagrande N. (2019). Formation of the Immunosuppressive Microenvironment of Classic Hodgkin Lymphoma and Therapeutic Approaches to Counter It. Int. J. Mol. Sci..

[40] Levine A.G., Mendoza A., Hemmers S., Moltedo B., Niec R.E., Schizas M., Hoyos B.E., Putintseva E.V., Chaudhry A., Dikiy S. (2017). Stability and function of regulatory T cells expressing the transcription factor T-bet. Nature.

[41] Wein F., Weniger M.A., Höing B., Arnolds J., Hüttmann A., Hansmann M.L., Küppers R. (2017). Complex Immune Evasion Strategies in Classical Hodgkin Lymphoma. Cancer Immunol. Res..

[42] Schreck S., Friebel D., Buettner M., Distel L., Grabenbauer G., Young L.S., Niedobitek G. (2009). Prognostic impact of tumour-infiltrating Th2 and regulatory T cells in classical Hodgkin lymphoma. Hematol. Oncol..

[43] Duffield A.S., Ascierto M.L., Anders R.A., Taube J.M., Meeker A.K., Chen S., McMiller T.L., Phillips N.A., Haiying X., Ogurtsova A. (2017). Th17 immune microenvironment in Epstein-Barr virus-negative Hodgkin lymphoma: Implications for immunotherapy. Blood Adv..

[44] Aoki T., Chong L.C., Takata K., Milne K., Hav M., Colombo A., Chavez E.A., Nissen M., Wang X., Miyata-Takata T. (2020). Single-Cell Transcriptome Analysis Reveals Disease-Defining T-cell Subsets in the Tumor Microenvironment of Classic Hodgkin Lymphoma. Cancer Discov..

[45] Dehghani M., Kalani M., Golmoghaddam H., Ramzi M., Arandi N. (2020). Aberrant peripheral blood CD4+CD25+FOXP3+ regulatory T cells/T helper-17 number is associated with the outcome of patients with lymphoma. Cancer Immunol. Immunother..

[46] Pizzolo G., Vinante F., Chilosi M., Dallenbach F., Josimovic-Alasevic O., Diamantstein T., Stein H. (1990). Serum levels of soluble CD30 molecule (Ki-1 antigen) in Hodgkin’s disease: Relationship with disease activity and clinical stage. Br. J. Haematol..

[47] Liu Y., Sattarzadeh A., Diepstra A., Visser L., van den Berg A. (2014). The microenvironment in classical Hodgkin lymphoma: An actively shaped and essential tumor component. Semin. Cancer Biol..

[48] Scheeren F.A., Diehl S.A., Smit L.A., Beaumont T., Naspetti M., Bende R.J., Blom B., Karube K., Ohshima K., van Noesel C.J.M. (2008). IL-21 is expressed in Hodgkin lymphoma and activates STAT5: Evidence that activated STAT5 is required for Hodgkin lymphomagenesis. Blood.

[49] Shahrara S., Pickens S.R., Dorfleutner A., Pope R.M. (2009). IL-17 Induces Monocyte Migration in Rheumatoid Arthritis. J. Immunol..

[50] Chetaille B., Betucci F., Finetti P., Esterni B., Stamatoullas A., Picquenot J.M., Copin M.C., Morschhauser F., Casasnovas O., Petrella T. (2009). Molecular profiling of classical Hodgkin lymphoma tissues uncovers variations in the tumor microenvironment and correlations with EBV infection and outcome. Blood.

[51] Zeng H., Zhang R., Jin B., Chen L. (2015). Type 1 regulatory T cells: A new mechanism of peripheral immune tolerance. Cell Mol. Immunol..

[52] Morales O., Mrizak D., François V., Mustapha R., Miroux C., Depil S., Decouvelaere A.V., Lionne-Huyghe P., Auriault C., de Launoit Y. (2014). Epstein-Barr virus infection induces an increase of T regulatory type 1 cells in Hodgkin lymphoma patients. Br. J. Haematol..

[53] Chikuma S. (2017). CTLA-4, an Essential Immune-Checkpoint for T-Cell Activation. Curr. Top. Microbiol. Immunol..

[54] Patel S.S., Weirather J.L., Lipschitz M., Lako A., Chen P.H., Griffin G.K., Armand P., Shipp M.A., Rodig S.J. (2019). The microenvironmental niche in classic Hodgkin lymphoma is enriched for CTLA-4-positive T cells that are PD-1-negative. Blood.

[55] Liu W.R., Shipp M.A. (2017). Signaling pathways and immune evasion mechanisms in classical Hodgkin lymphoma. Blood.

[56] Jones K., Wockner L., Brennan R.M., Keane C., Chattopadhyay P.K., Roederer M., Price D.A., Cole D.K., Hassan B., Beck K. (2016). The impact of HLA class I and EBV latency-II antigen-specific CD8(+) T cells on the pathogenesis of EBV(+) Hodgkin lymphoma. Clin. Exp. Immunol..

[57] Gandhi M.K., Lambley E., Duraiswamy J., Dua U., Smith C., Elliott S., Gill D., Marlton P., Seymour J., Khanna R. (2006). Expression of LAG-3 by tumor-infiltrating lymphocytes is coincident with the suppression of latent membrane antigen-specific CD8+ T-cell function in Hodgkin lymphoma patients. Blood.

[58] van der Leun A.M., Thommen D.S., Schumacher T.N. (2020). CD8+ T cell states in human cancer: Insights from single-cell analysis. Nat. Rev. Cancer.

[59] Le K.S., Amé-Thomas P., Tarte K., Gondois-Rey F., Granjeaud S., Orlanducci F., Foucher E.D., Broussais F., Bouabdallah R., Fest T. (2018). CXCR5 and ICOS expression identifies a CD8 T-cell subset with T_FH_ features in Hodgkin lymphomas. Blood Adv..

[60] Silina K., Soltermann A., Attar F.M., Casanova R., Uckeley Z.M., Thut H., Wandres M., Isajevs S., Cheng P., Curioni-Fontecedro A. (2018). Germinal Centers Determine the Prognostic Relevance of Tertiary Lymphoid Structures and Are Impaired by Corticosteroids in Lung Squamous Cell Carcinoma. Cancer Res..

[61] Gandhi M.K., Moll G., Smith C., Dua U., Lambley E., Ramuz O., Devinder G., Marlton P., Seymour J.F., Khanna R. (2007). Galectin-1 mediated suppression of Epstein-Barr virus-specific T-cell immunity in classic Hodgkin lymphoma. Blood.

[62] O’Connor G.M., Hart O.M., Gardiner C.M. (2005). Putting the natural killer cell in its place. Immunology.

[63] Reichel J., Chadburn A., Rubinstein P.G., Giulino-Roth L., Tam W., Liu Y., Gaiolla R., Eng K., Brody J., Inghirami G. (2015). Flow sorting and exome sequencing reveal the oncogenome of primary Hodgkin and Reed-Sternberg cells. Blood.

[64] Chiu J., Ernst D.M., Keating A. (2018). Acquired Natural Killer Cell Dysfunction in the Tumor Microenvironment of Classic Hodgkin Lymphoma. Front. Immunol..

[65] Schröder M., Meisel C., Buhl K., Profanter N., Sievert N., Volk H.D., Grütz G. (2003). Different Modes of IL-10 and TGF-β to Inhibit Cytokine-Dependent IFN-γ Production: Consequences for Reversal of Lipopolysaccharide Desensitization. J. Immunol..

[66] Gooding R., Riches P., Dadian G., Moore J., Gore M. (1995). Increased soluble interleukin-2 receptor concentration in plasma predicts a decreased cellular response to IL-2. Br. J. Cancer..

[67] Medvedev A.E., Johnsen A.C., Haux J., Steinkjer B., Egeberg K., Lynch D.H., Sundan A., Espevik T. (1997). Regulation of Fas and Fas-ligand expression in NK cells by cytokines and the involvement of Fas-ligand in NK/LAK cell-mediated cytotoxicity. Cytokine.

[68] Carey C.D., Gusenleitner D., Lipschitz M., Roemer M.G.M., Stack E.C., Gjini E., Hu X., Redd R., Freeman G.J., Neuberg D. (2017). Topological analysis reveals a PD-L1-associated microenvironmental niche for Reed-Sternberg cells in Hodgkin lymphoma. Blood.

[69] Cooper M.A., Fehniger T.A., Caligiuri M.A. (2001). The biology of human natural killer-cell subsets. Trends Immunol..

[70] Vari F., Arpon D., Keane C., Hertzberg M.S., Talaulikar D., Jain S., Cui Q., Han E., Tobin J., Bird R. (2018). Immune evasion via PD-1/PD-L1 on NK cells and monocyte/macrophages is more prominent in Hodgkin lymphoma than DLBCL. Blood.

[71] Stannard K.A., Lemoine S., Waterhouse N.J., Vari F., Chatenoud L., Gandhi M.K., Martinet L., Smyth M.J., Guillerey C. (2019). Human peripheral blood DNAM-1 neg NK cells are a terminally differentiated subset with limited effector functions. Blood Adv..

[72] Lee C.W., Ren Y.J., Marella M., Wang M., Hartke J., Couto S.S. (2020). Multiplex immunofluorescence staining and image analysis assay for diffuse large B cell lymphoma. J. Immunol. Methods.

[73] Baharlou H., Canete N.P., Cunningham A.L., Harman A.N., Patrick E. (2019). Mass Cytometry Imaging for the Study of Human Diseases-Applications and Data Analysis Strategies. Front. Immunol..

[74] Reinke S., Bröckelmann P.J., Iaccarino I., Garcia-Marquez M.A., Borchmann S., Jochims F., Kotrova M., Pal K., Brüggemann M., Hartmann E. (2020). Tumor and microenvironment response but no cytotoxic T-cell activation in classic Hodgkin lymphoma treated with anti-PD1. Blood.

[75] Veldman J., Visser L., Huberts-Kregel M., Muller N., Hepkema B., Van den Berg A., Diepstra A. (2020). Rosetting T cells in Hodgkin lymphoma are activated by immunological synapse components HLA class II and CD58. Blood.

[76] Boussiotis V.A. (2016). Molecular and Biochemical Aspects of the PD-1 Checkpoint Pathway. N. Engl. J. Med..

[77] Pentcheva-Hoang T., Egen J.G., Wojnoonski K., Allison J.P. (2004). B7-1 and B7-2 Selectively Recruit CTLA-4 and CD28 to the Immunological Synapse. Immunity.

[78] Long L., Zhang X., Chen F., Pan Q., Phiphatwatchara P., Zeng Y., Chen H. (2018). The promising immune checkpoint LAG-3: From tumor microenvironment to cancer immunotherapy. Genes Cancer.

[79] Jalali S., Price-Troska T., Bothun C., Villasboas J., Kim H.J., Yang Z.Z., Novak A.J., Dong H., Ansell S.M. (2019). Reverse signaling via PD-L1 supports malignant cell growth and survival in classical Hodgkin lymphoma. Blood Cancer J..

[80] Ansell S.M., Lesokhin A.M., Borrello I., Halwani A., Scott E.C., Gutierrez M., Schuster S.J., Millenson M.M., Cattry D., Freeman G.J. (2015). PD-1 Blockade with Nivolumab in Relapsed or Refractory Hodgkin’s Lymphoma. N. Engl. J. Med..

[81] Armand P., Shipp M.A., Ribrag V., Michot J., Zinzani P.L., Kuruvilla J., Snyder E.S., Ricart A.D., Balakumaran A., Rose S. (2016). Programmed Death-1 Blockade with Pembrolizumab in Patients with Classical Hodgkin Lymphoma After Brentuximab Vedotin Failure. J. Clin. Oncol..

[82] Armand P., Engert A., Younes A., Fanale M., Santoro A., Zinzani P.L., Timmerman J.M., Collins G.P., Ramchandren R., Cohen J.B. (2018). Nivolumab for Relapsed/Refractory Classic Hodgkin Lymphoma After Failure of Autologous Hematopoietic Cell Transplantation: Extended Follow-Up of the Multicohort Single-Arm Phase II CheckMate 205 Trial. J. Clin. Oncol..

[83] Chen R., Zinzani P.L., Lee H.J., Armand P., Johnson N.A., Brice P., Radford J., Ribrag V., Molin D., Vassilakopoulos T.P. (2019). Pembrolizumab in relapsed or refractory Hodgkin lymphoma: 2-year follow-up of KEYNOTE-087. Blood.

[84] Roemer M.G.M., Redd R.A., Cader F.Z., Pak C.J., Abdelrahman S., Ouyang J., Sasse S., Younes A., Fanale M., Santoro A. (2018). Major Histocompatibility Complex Class II and Programmed Death Ligand 1 Expression Predict Outcome After Programmed Death 1 Blockade in Classic Hodgkin Lymphoma. J. Clin. Oncol..

[85] Derenzini E., Lemoine M., Buglio D., Katayama H., Ji Y., Davis R.E., Sen S., Younes A. (2011). The JAK inhibitor AZD1480 regulates proliferation and immunity in Hodgkin lymphoma. Blood Cancer J..

[86] Nagasaki J., Togashi Y., Sugawara T., Itami M., Yamauchi N., Yuda J., Sugano M., Ohara Y., Minami Y., Nakamae H. (2020). The critical role of CD4+ T cells in PD-1 blockade against MHC-II–expressing tumors such as classic Hodgkin lymphoma. Blood Adv..

[87] Cader F.Z., Hu X., Goh W.L., Wienand K., Ouyang J., Mandato E., Redd R., Lawton L.N., Chen P.H., Weirather J.L. (2020). A peripheral immune signature of responsiveness to PD-1 blockade in patients with classical Hodgkin lymphoma. Nat. Med..

[88] Kline J., Godfrey J., Ansell S.M. (2020). The immune landscape and response to immune checkpoint blockade therapy in lymphoma. Blood.

[89] Koyama S., Akbay E.A., Li Y.Y., Herter-Sprie G.S., Buczkowski K.A., Richards W.G., Gandhi L., Redig A.J., Rodig S.J., Asahina H. (2016). Adaptive resistance to therapeutic PD-1 blockade is associated with upregulation of alternative immune checkpoints. Nat. Commun..

[90] Pauken K.E., Sammons M.A., Odorizzi P.M., Manne S., Godec J., Khan O., Drake A.M., Chen Z., Sen D.R., Kurachi M. (2016). Epigenetic stability of exhausted T cells limits durability of reinvigoration by PD-1 blockade. Science.

[91] Ghoneim H.E., Fan Y., Moustaki A., Abdelsamed H.A., Dash P., Dogra P., Carter R., Awad W., Neale G., Thomas P.G. (2017). De Novo Epigenetic Programs Inhibit PD-1 Blockade-Mediated T Cell Rejuvenation. Cell.

[92] Nie J., Wang C., Liu Y., Yang Q., Mei Q., Dong L., Li X., Liu J., Ku W., Zhang Y. (2019). Addition of Low-Dose Decitabine to Anti-PD-1 Antibody Camrelizumab in Relapsed/Refractory Classical Hodgkin Lymphoma. J. Clin. Oncol..

[93] Armand P., Lesokhin A., Borrello I., Timmerman J., Gutierrez M., Zhu L., Popa McKiver M., Ansell S.M. (2020). A phase 1b study of dual PD-1 and CTLA-4 or KIR blockade in patients with relapsed/refractory lymphoid malignancies. Leukemia.

[94] Rodig S.J., Gusenleitner D., Jackson D.G., Gjini E., Giobbie-hurder A., Jin C., Jin C., Chang H., Lovitch S.B., Horak C. (2018). MHC proteins confer differential sensitivity to CTLA-4 and PD-1 blockade in untreated metastatic melanoma. Sci. Transl. Med..

[95] Burova E., Hermann A., Dai J., Ullman E., Halasz G., Potocky T., Hong S., Liu M., Allbritton O., Woodruff A. (2019). Preclinical Development of the Anti-LAG-3 Antibody REGN3767: Characterization and Activity in Combination with the Anti-PD-1 Antibody Cemiplimab in Human PD-1xLAG-3-Knockin Mice. Mol. Cancer Ther..

[96] Yu X., Huang X., Chen X., Liu J., Wu C., Pu Q., Wang Y., Kang X., Zhou L. (2019). Characterization of a novel anti-human lymphocyte activation gene 3 (LAG-3) antibody for cancer immunotherapy. MAbs.

[97] Ascierto P.A., Melero I., Bhatia S., Bono P., Sanborn R.E., Lipson E.J., Callahan M.K., Gajewski T., Gomez-Roca C.A., Hodi F.S. (2017). Initial efficacy of anti-lymphocyte activation gene-3 (anti–LAG-3; BMS-986016) in combination with nivolumab (nivo) in pts with melanoma (MEL) previously treated with anti–PD-1/PD-L1 therapy. J. Clin. Oncol..

[98] Rothe A., Sasse S., Topp M.S., Eichenauer D.A., Hummel H., Reiners K.S., Dietlein M., Kuhnert G., Kessler J., Buerkle C. (2015). A phase 1 study of the bispecific anti-CD30/CD16A antibody construct AFM13 in patients with relapsed or refractory Hodgkin lymphoma. Blood.

[99] Bartlett N.L., Herrera A.F., Domingo-Domenech E., Mehta A., Forero-Torres A., Garcia-Sanz R., Armand P., Devata S., Rodriguez Izquierdo A., Lossos I.S. (2020). A phase 1b study of AFM13 in combination with pembrolizumab in patients with relapsed or refractory Hodgkin lymphoma. Blood.

[100] Janik J.E., Morris J.C., O’Mahony D., Pittaluga S., Jaffe E.S., Redon C.E., Bonner W.M., Brechbiel M.W., Paik C.H., Whatley M. (2015). 90Y-daclizumab, an anti-CD25 monoclonal antibody, provided responses in 50% of patients with relapsed Hodgkin’s lymphoma. Proc. Natl. Acad. Sci. USA.

[101] Hamadani M., Collins G.P., Samaniego F., Spira A.I., Davies A., Radford J., Caimi P., Menne T., Boni J., Cruz H. (2018). Phase 1 Study of Adct-301 (Camidanlumab Tesirine), a Novel Pyrrolobenzodiazepine-Based Antibody Drug Conjugate, in Relapsed/Refractory Classical Hodgkin Lymphoma. Blood.

[102] Roswarski J., Roschewski M., Lucas A., Melani C., Pittaluga S., Jaffe E.S., Steinberg S.M., Waldmann T.A., Wilson W.H. (2018). Phase I dose escalation study of the anti-CD2 monoclonal antibody, siplizumab, with DA-EPOCH- R in aggressive peripheral T-cell lymphomas. Leuk. Lymphoma.

[103] Diefenbach C.S., Hong F., Ambinder R.F., Cohen J.B., Robertson M.J., David K.A., Advani R.H., Fenske T.S., Barta S.K., Palmisiano N.D. (2020). Ipilimumab, nivolumab, and brentuximab vedotin combination therapies in patients with relapsed or refractory Hodgkin lymphoma: Phase 1 results of an open-label, multicentre, phase 1/2 trial. Lancet Haematol..

[104] Hansen H.P., Engels H.M., Dams M., Paes Leme A.F., Pauletti B.A., Simhadri V.L., Dürkop H., Reiners K.S., Barnert S., Engert A. (2014). Protrusion-guided extracellular vesicles mediate CD30 trans-signalling in the microenvironment of Hodgkin’s lymphoma. J. Pathol..

[105] Ramos C.A., Grover N.S., Beaven A.W., Lulla P.D., Wu M., Ivanova A., Wang T., Shea T.S., Rooney C.M., Dittus C. (2020). Anti-CD30 CAR-T Cell Therapy in Relapsed and Refractory Hodgkin Lymphoma. J. Clin. Oncol..

